# A Systematic Review and Meta-Analysis of B Vitamin Supplementation on Depressive Symptoms, Anxiety, and Stress: Effects on Healthy and ‘At-Risk’ Individuals

**DOI:** 10.3390/nu11092232

**Published:** 2019-09-16

**Authors:** Lauren M Young, Andrew Pipingas, David J White, Sarah Gauci, Andrew Scholey

**Affiliations:** Centre for Human Psychopharmacology, Swinburne University, Melbourne, VIC 3122, Australia; laurenyoung@swin.edu.au (L.M.Y.); apipingas@swin.edu.au (A.P.); dawhite@swin.edu.au (D.J.W.); sarahgauci@swin.edu.au (S.G.)

**Keywords:** B-vitamins, mood, meta-analysis, review, mental health, stress, anxiety, depression

## Abstract

A systematic review and meta-analysis was undertaken to examine and quantify the effects of B vitamin supplementation on mood in both healthy and ‘at-risk’ populations. A systematic search identified all available randomised controlled trials (RCTs) of daily supplementation with ≥3 B group vitamins with an intervention period of at least four weeks. Random effects models for a standardized mean difference were used to test for overall effect. Heterogeneity was tested using the I^2^ statistic. Eighteen articles (16 trials, 2015 participants) were included, of which 12 were eligible for meta-analysis. Eleven of the 18 articles reported a positive effect for B vitamins over a placebo for overall mood or a facet of mood. Of the eight studies in ‘at-risk’ cohorts, five found a significant benefit to mood. Regarding individual facets of mood, B vitamin supplementation benefited stress (*n* = 958, SMD = 0.23, 95% CI = 0.02, 0.45, *p* = 0.03). A benefit to depressive symptoms did not reach significance (*n* = 568, SMD = 0.15, 95% CI = −0.01, 0.32, *p* = 0.07), and there was no effect on anxiety (*n* = 562, SMD = 0.03, 95% CI = −0.13, 0.20, *p* = 0.71). The review provides evidence for the benefit of B vitamin supplementation in healthy and at-risk populations for stress, but not for depressive symptoms or anxiety. B vitamin supplementation may particularly benefit populations who are at risk due to (1) poor nutrient status or (2) poor mood status.

## 1. Introduction

Mood is an emotional mental state that can fluctuate both acutely and over time. An individual’s experience of mood is dependent on both exogenous ‘situational’ factors and intrinsic ‘dispositional’ factors, which are assumed to involve endogenous physiological processes such as fluctuations in hormones, neurotransmitters, and nutrients. Within psychiatry, there are clinical thresholds for the diagnosis of mood disorders. The Global Burden of Disease Study 2010 reported that 18.9% of all years lived with a disability could be attributed to mental disorders [1]. Conditions such as depression, dysthymia, and anxiety place a large burden on the health care system since they account for a significant proportion of the world’s disease burden [1]. These figures do not include those with symptomatology, which does not reach the threshold for clinical diagnosis but, nevertheless, may be problematic for the individuals and may signal a heightened risk for a psychiatric disorder. Clearly, there are also natural variations in mood within the healthy population, including within some individuals who are predisposed to such disorders [2]. Optimising mood and well-being in non-clinical populations may, therefore, represent a realistic method for preventing a clinical disorder. A shift in research focus for understanding how mood may be optimised in healthy and ‘at-risk’ populations is warranted.

Over the past two decades, the influence of diet on brain health has been considered as a modifiable risk factor to prevent mood disorder. Nutritional interventions designed to improve diet quality have been reported to reduce depressive symptoms, independent of self-efficacy and physical activity levels [3]. A number of observational studies indicate a relationship between a poor diet and worse mental health [4,5]. Many of the foods considered to improve “diet quality,” and, by implication, mood, resemble the foods from the “Mediterranean Diet.” These include lean meats, fish, green leafy vegetables, legumes, and nuts. Of relevance to this review, such food groups are rich in B-group vitamins raising the possibility that the relationship between the mood and dietary habits may be mediated, in part, by intake of these micronutrients. B vitamin status has purported a benefit to brain health and mood [6,7,8]. Deficiencies in these micronutrients, such as B12 or folate, are associated with increased risk and incidence of depression [9]. This has led to calls for increased recognition of nutritional deficiency, and/or suboptimal nutrient status, as contributing to the underlying causes of mood disturbances and other psychiatric conditions [10,11].

The integral role of B vitamins as cofactors in cellular processes such as the methionine and folate cycles have formed the basis for hypotheses relating B vitamin status with mood [12]. Vitamins B6, B12, and folate are commonly acknowledged as cofactors for enzymatic reactions in the methionine and folate cycles. Kennedy [6] also highlights that the full range of B vitamins contribute to the interlinked cellular processes responsible for DNA methylation and clearance of homocysteine. Thus, deficiencies in one or a number of these nutrients may limit these metabolic pathways, which results in the accumulation of homocysteine [13]. The hypothesis that hyper-homocysteinaemia is a risk factor for poor mood is strengthened by a study reporting that up to 30% of depressed patients have elevated homocysteine levels [14]. In this regard, one would expect that supplementation with B group vitamins designed to lower homocysteine would support benefits to mood outcomes. 

Through their role in one-carbon metabolism, B vitamins act as cofactors in the synthesis and regulation of dopaminergic and serotonergic neurotransmitters. Both of these neurotransmitters are implicated in the regulation of mood, as well as clinical depression and anxiety. As such, both are common targets for antidepressant medication. B vitamin supplementation may, therefore, offer an alternative or adjunctive treatment to standard care aimed at optimising mood via modulation of the neurotransmitter function. Such an approach may have a lower risk of adverse side effects compared with current antidepressants [15].

Many studies have investigated the mood effects of B vitamins in clinically depressed, medicated participants [15,16]. Subsequently, it can be difficult to unpick the direct effects of supplementation from those resulting from interactions with anti-depressants. Clearly, such studies provide useful information regarding the effectiveness of B vitamins for this population as adjunctive therapy to medication. However, they tell us less about the potential benefits of B vitamin supplementation *per se*. Furthermore, there may be structural and functional differences in the neural architecture of participants with depression [17,18], which make it difficult to generalise any mood benefits to non-clinical populations.

A further issue is that research into B vitamin supplementation has typically used multi-nutrient interventions containing a number of other vitamins and minerals. Thus, the interaction of B vitamins with these other nutrients needs to be considered. Laville, et al. [19] suggest that the baseline nutrient status may affect the response to a nutrient intervention, possibly due to idiosyncratic effects of individual’s gastrointestinal microbiota. There is also increasing consensus that the suboptimal nutrient status, which does not meet criteria for frank clinical deficiency, may be a risk factor for a poor functional status [11,20]. The emerging field of nutritional cognitive neuroscience includes studies relating central structure and function to specific ‘nutrient biomarker patterns’ in the context of neurocognitive health [21,22,23]. While it is important to determine whether individual B vitamins are effective, it is also critical to consider these findings in the context of the baseline nutrient status, dietary habits, and co-administration of other nutrients as part of the intervention.

Lastly, there are a number of Randomized Controlled Trials (RCTs) that have examined the effects of individual B group vitamins on mood regulation [24,25,26,27]. These have typically been limited to clinical populations and have produced equivocal findings. Kennedy [6] argues that this may be due to the interdependence of B group vitamins within the methylation cycle. Therefore, a combination of B group vitamins should be more effective than supplementation with B6, B12, or folate alone, as, in situ, these micronutrients operate in concert to carry out physiological processes [28]. This is supported to some degree by a previous meta-analysis reporting that multi-nutrient supplements, which contained relatively higher doses of B vitamins, were more effective in improving mood in healthy populations [8]. Unfortunately, the mood effects of broad spectrum B group vitamins have been less well scrutinized, in both clinical and healthy populations.

Given the increasing number of individuals afflicted by mental health problems, there is an imperative need to understand preventative measures to reduce the incidence of mental illness. Moreover, it appears that nutrient status and function are on a continuum. Following the recommendations of Monti, et al. [29], participants who are most likely to benefit from intervention were selected. In this case, individuals with a suboptimal nutrient status were selected, which would enhance the potential to detect any effect. Targeting individuals who are clinically intact but considered ‘at-risk’ may allow for B vitamins to be used as a preventative method from progressing along a continuum toward clinical disorders.

It is important to note that the Recommended Daily Allowances (RDAs) for micronutrients were developed as guidelines to prevent risk of disease. These frameworks were never created in the context of optimizing physical or psychological health [30]. Further research is required to understand if B vitamin supplementation is an appropriate method to improve mood in non-clinical or at-risk populations. This is particularly important in light of evidence that improving nutrient status may partially mitigate risk of future mood and cognitive disorders [11,23].

The present review examines the effects of B vitamin supplementation on mood outcomes in randomized, placebo-controlled trials in both healthy and ‘at risk’ populations. Specifically, it aims to address the extent to which the current literature supports any benefit of taking B vitamins for mood in both healthy and at-risk samples.

Two previous reviews are particularly relevant to the approach taken in this case. First, Long and Benton [8] reported that trials utilising multivitamin/mineral supplements that were more rich in B vitamins resulted in greater improvements in perceived stress and psychiatric symptoms in non-clinical samples. Thus, the present review will be restricted to the effects of B vitamin supplementation, regardless of whether the vitamins are provided within a multivitamin/mineral or as an exclusively B-group vitamin combination. Second, Kennedy’s [6] review of B vitamin effects on cognitive mechanisms made a convincing case for the effects of multiple B-group vitamins being greater than the effect of individual B vitamins. Accordingly, the present document is restricted to studies that have utilized broad spectrum B vitamins (≥3), rather than single nutrients.

This review was performed in order to (1) explore a broader range of facets of mood to include stress, as seen in the Long and Benton [8], (2) understand how nutrient status may impact mood through the analysis of blood biomarkers when these data are available and (3) consider how B vitamin supplementation may benefit at-risk populations in comparison to healthy populations.

## 2. Materials and Methods 

### 2.1. Searches

Medline (PubMed and Web of Science), Scopus, and PsycINFO were searched for randomized controlled trials until January 2019 by combining terms relating to the intervention (“B vitamins,” “vitamin B,” “vitamin B complex,” “vitamin B12,” “vitamin B6,” “folate,” “cobalamin,” “folic acid,” “multivitamin,” “multi-nutrient”), terms relating to the mood outcome (“mood,” “depress,*” “anxiety,” “affect,*” “stress,*” “fatigue,” and “mood disorder” and terms relating to the study design (“randomised control trial,” “randomized control trial,” “randomised controlled trial,” “randomized controlled trial,” and “clinical trial”). When possible, search results were automatically filtered using the following limits: human studies, adult samples, and published 2000 onward. In addition, further studies were examined based on examination of reference lists from relevant studies.

### 2.2. Eligibility Criteria

The primary focus of this review was to evaluate the effects of B vitamin supplementation on mood. In accordance with Preferred Reporting Items for Systematic Reviews and Meta-Analyses (PRISMA) guidelines, to limit heterogeneity between the included studies, the following inclusion criteria were used. To be considered for selection, trials had to be randomized, double-blind, placebo-controlled trials and had to report on a mood outcome (including stress, well-being, and fatigue). Additionally, the mood outcome must have been quantifiable, which means any change could be measured over the course of the intervention period. Studies that examined incidence of clinical mood disorders were excluded.

Studies must involve a daily supplement containing ≥3 B group vitamins with an intervention period of at least four weeks. This deviates from the review by Long and Benton [8] that set criteria aiming to exclude “studies of two or three B vitamins that aimed to decrease homocysteine levels.” Studies included in the present review may have given a supplement containing other vitamins and/or minerals, or a supplement that contained B group vitamins only. The rationale to include at least three B group vitamins was made on the basis of Kennedy [6], who concluded that the effect of multiple B group vitamins were more effective than single nutrients.

Only studies involving non-clinical adult populations (>18 years of age) were included. Studies that included participants who were ‘at-risk’ of mood disorders were included in addition to healthy populations. To meet ‘at-risk’ criteria, the studied population must have been considered a vulnerable group, i.e., having a nutritional deficiency, elevated levels of a psychological mood measure, sub-clinical symptomatology or vulnerability to mood disorders. The eligibility section of these studies was carefully considered to ensure participants were, otherwise, free from illness and did not have a clinical diagnosis of a mood disorder.

While blood biomarkers of B vitamin levels were of interest, studies were not excluded on the basis of whether or not they measured or reported biomarker status.

Studies published from 2000 onward only were included.

### 2.3. Study Selection and Data Extraction

**Figure 1:**
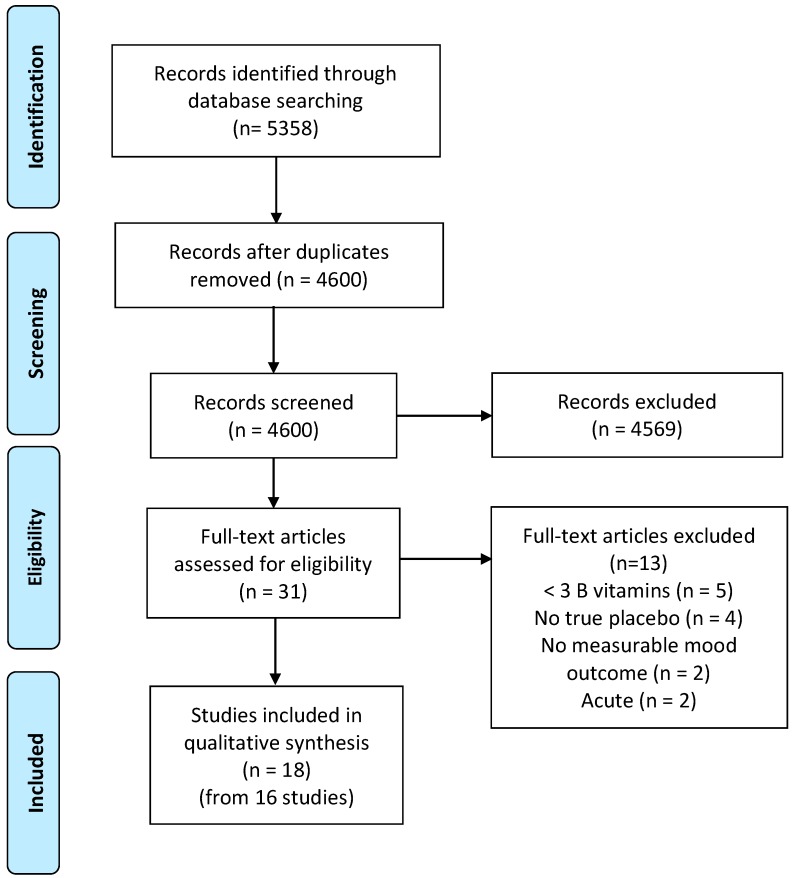
Flow diagram depicting the selection process for articles included in this meta-analysis.

One reviewer (LY) conducted the article search, removed duplicates, and screened papers by titles and abstracts using the methodology of Rayyan [31]. Papers undergoing full text evaluation were appraised by two reviewers (LY and SG). The following articles in this review were agreed on by both the researchers. Figure 1 outlines the selection process.

Data extraction was completed by two reviewers (LY and SG) once each study had been assessed against inclusion criteria.

Study duration, sample demographics (age, gender), and sample sizes were extracted. In the event of multiple armed studies, only data for supplements containing B-group vitamins and the placebo group were extracted. In the event of multiple time points, only values concerning baseline and end-point data were extracted. The type, version, and nature of questionnaires used was recorded. The composition of the daily dose of B vitamins for each supplement was also extracted. Reviewers made note of whether there was any objective measure of B-vitamin intake such as blood biomarkers, or a measure of dietary intake of B vitamins. If present, reviewers extracted the baseline and follow-up means and standard deviations of these measures. If there was no mention of these measures in the Methods section of the article, it was assumed that no measure was obtained.

### 2.4. Data Analyses

Mood outcomes were categorised as measures of ‘stress,’ ‘depressive symptoms,’ or ‘anxiety symptoms.’ Twelve of the 18 studies were deemed eligible for meta-analysis. Effect sizes were calculated by subtracting the mean change for the placebo group from the mean change in the B vitamin group and dividing by the pooled standard deviation at baseline.

(1) $$ES=\;\frac{{\bar{x}}_{1}-\;{\bar{x}}_{2}}{s}$$

Meta-analysis was conducted using Cochrane Review Manager Software, RevMan Review Manager 5.3, Copenhagen, Denmark [32]. In all analyses, random effects model for a standardized mean difference was used to test for the overall effect. Heterogeneity was tested using I^2^ statistic on all models and assessed according to guidelines outlined by Shamseer, et al. [33]. I^2^ values of 0%–40% might not be important. In addition, 30%–60% may represent moderate heterogeneity, 50%–90% may represent substantial heterogeneity, and 75%–100% may represent considerable heterogeneity. If considerable heterogeneity was observed (I^2^ ≥ 75%), funnel plots were inspected to identify possible sources of heterogeneity and a decision was made on whether outliers should be removed to increase homogeneity to acceptable levels [33].

In the case of a study reporting common data from multiple measures, only one measure was used so that participants were not represented twice within the same analyses. Where two or more such measures were reported, the chosen outcome used in the meta-analysis was based solely on the basis of how frequently it had been reported across studies, to assist homogeneity.

## 3. Results

### 3.1. Study Selection

The initial search captured 5358 records. After duplicates were removed, 4600 titles and abstracts were screened for potentially relevant RCTs, with the full text of 31 studies assessed for their eligibility. Eighteen articles (from 16 trials) fulfilled the inclusion criteria and were included in this review. Figure 1 outlines the search process and reasons for exclusion. Table 1 outlines the studies included in this review.

Two pairs of articles were identified as using different data from the same cohort of participants, Kennedy, et al. [34] and Kennedy, et al. [35], Camfield, et al. [36] and Pipingas, et al. [37]. After accounting for these, there was a total of 2015 participants across 16 trials. Eight studies included healthy participants, and eight utilized samples that may be considered ‘at-risk.’ These samples consisted of highly stressed adult employees [38], celiac patients [39], nursing home residents [40], individuals being treated for, or with a history of hypertension [41], having occasional subjective fatigue [42], chronic psychological stress [43], elevated psychological distress [44], and holding a sedentary occupation or leading a sedentary lifestyle [45]. Importantly, these participants did not have a clinical diagnosis of a mood disorder and were, otherwise, physically healthy. The length of the intervention period ranged from 28 days to two years. Fourteen trials included a B vitamin as part of a broader spectrum multi-vitamin. Two studied the use of B-group supplements alone, containing B6, B12, and folate [39,41]. Five studies included male participants only, three studies included only female participants, and the remaining studies recruited both male or female participants, or was unspecified. Only two of the included studies reported measuring baseline dietary habits in the form of a questionnaire [46] and three-day food records [43].

### 3.2. Constituents of Supplements

Two studies considered B vitamins alone [39,41] while all of the included studies used multi-vitamin supplements that contained up to eight B group vitamins (see Table 2).

All of the included studies used supplements containing B6 and B12, with all but one study including folate. Vitamins B1, B2, B3, and B5 were included in 16 of the 18 included studies. Vitamin B7 was the least included micro-nutrient and was only included in 10 studies.

In comparison with the recommended daily intakes set by the Australian National Health and Medical Research Council (NHMRC), most supplements contained double the recommended daily intake of B vitamins, with some exceeding the daily intake by 10 to 300 times. An exception to this is the supplement used by Long and Benton [47], which contained levels similar to the recommended doses.

In terms of other constituents in the supplement, two studies included B vitamins only. Seven studies included three to four other constituents, with the remaining containing a higher number of micronutrients or botanical constituents. The largest amount of other constituents came from Macpherson, et al. [49], with a total of 37 additional constituents.

The following section summarizes the effects of supplementation on broad measure of overall mood and specific measures of depression, stress, and anxiety. It should be noted that each specific outcome is either a direct measure of stress, depressive symptoms, or anxiety, or is derived from a subscale within the broader measures of mood. A positive effect for overall mood or a facet of mood was reported in 11 of 18 articles over the effect of the placebo. These are summarized in Table 1.

### 3.3. Overall Mood

Eleven of the included studies utilized at least one broad measure of overall mood, such as a measure of mental health or current state of psychological well-being. Of the five studies that utilized the General Health Questionnaire (GHQ), three found positive effects of B vitamin supplementation on overall mental well-being, while the remaining two studies saw no effect. Five studies that used the Profile of Mood States (POMS) found no effects of supplementation on total mood disturbance, with one study finding a trending benefit [34].

Harris et al. [45] reported an overall reduction in negative mood symptoms on the Depression, Anxiety, and Stress Scale. Hallert et al. [39] found benefits of supplementation as measured by the Psychological General Well-Being Index in a subgroup of participants only, i.e., those who had a poorer mood at baseline. Due to a number of studies measuring “overall mood” with more than one measure, each composed of a number of sub-scales. Meta-analysis of ‘overall mood’ was not reported since it was better captured in mood facets in the following sections.

### 3.4. Depressive Symptoms

Depression was the most widely studied mood dimension, with a measure of depression included in 11 of the 18 included articles. However, there was large heterogeneity between measures, with a total of seven different measures of depression. Of the five studies that used the POMS depression-dejection scale, only White, et al. [50] found evidence for a benefit from B vitamins, notably after a much shorter intervention period than some of the other studies (28 days). Two other studies found a benefit of B vitamin supplementation for depressive symptoms in a subgroup of their sample. Those participants have a poor depression score at baseline [39,40]. In contrast, Ford, Flicker, Thomas, Norman, Jamrozik and Almeida [41] conducted a similar analysis, finding no significant effect for the entire cohort, or for those participants with higher depressive symptoms at baseline. 

**Figure 2:**
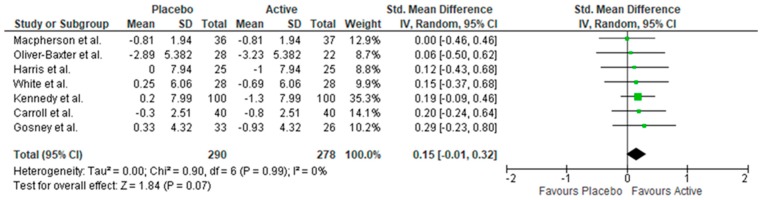
Forest plot of meta-analysis of depressive symptoms.

Meta-analysis was conducted on nine studies measuring depressive symptoms, which revealed no benefit of supplementation (see Appendix A). The I^2^ statistic indicated considerable heterogeneity (I^2^ = 95%, *p* < 0.01). Inspection of funnel plots revealed two extreme outliers (one favouring placebo, one favouring supplementation). Upon removing outliers, the test was homogenous (I^2^ = 0%, *p* < 0.99). Meta-analysis revealed that supplementation reduced depressive symptoms. However, the test failed to reach significance (*n* = 568, SMD = 0.15, 95% CI = −0.01, 0.32, *p* = 0.07) (Figure 2).

### 3.5. Stress

**Figure 3:**
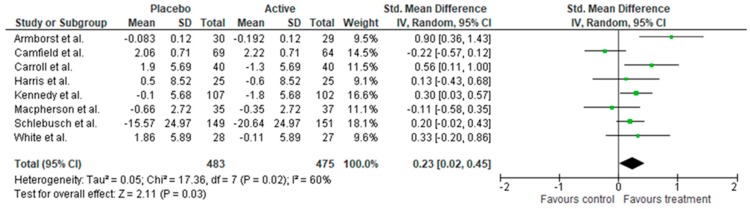
Forest plot of meta-analysis on stress symptoms.

A measure of stress was included in 10 studies, with seven of these studies utilising the Perceived Stress Scale (PSS). Three of these found positive effects of supplementation by decreasing subjective stress. An additional three studies found benefits of B vitamin supplementation on stress using the BSI and PSQ [38,43,51]. In total, six of the 10 studies measuring stress found a benefit of supplementation. Meta-analysis revealed supplementation reduced stress symptoms (*n* = 958, SMD = 0.23, 95% CI = 0.02, 0.45, *p* = 0.03) (Figure 3). However, there was moderate heterogeneity across measures (I^2^ = 60%) and chi-square was violated (*p* = 0.02).

### 3.6. Anxiety Symptoms

**Figure 4:**
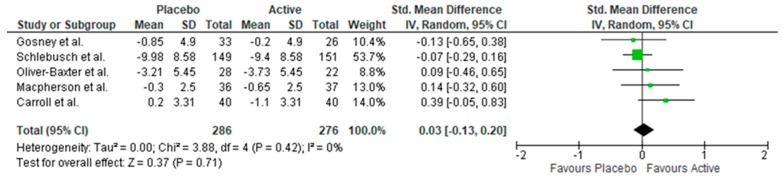
Forest plot of meta-analysis on anxiety symptoms.

Ten studies included a measure of anxiety. Two studies found benefits to anxiety symptoms following short supplementation periods (28–30 days) ([38] and [46]). Another study found a benefit of supplementation in a sub-group of participants who had a poor anxiety status at baseline [39]. Lastly, Pipingas, Camfield, Stough, Cox, Fogg, Tiplady, Sarris, White, Sali, Wetherell and Scholey [37] found a significant benefit of supplementation in a male subgroup only. The remaining studies found no benefit of B vitamins over a placebo. Meta-analysis revealed supplementation had no benefit for stress symptoms (*n* = 562, SMD = 0.03, 95% CI = −0.13, 0.20, *p* = 0.71, see Figure 4).

### 3.7. Biomarkers

Of the 16 trials, seven collected information regarding blood biomarkers as a measure of B vitamin status and its change with supplementation. Folate was the most widely measured (seven studies). Four studies reported B12 levels, three studies reported B6 levels, and one study reported B1 and B2 levels [52]. Homocysteine was also measured in five studies as a mechanism.

Assessing biomarkers at baseline provides an opportunity to reveal the nutritional status of participants selected for the studies. Table 3 displays the mean biomarker levels of B vitamins and homocysteine measured at baseline. No study reported a mean level that was consistent with clinical deficiency at baseline. Of the studies measuring homocysteine, three studies were well within the normal range (<15 µmol/L). However, the two other studies measuring homocysteine had higher baseline levels, with participants within one standard deviation of the mean considered in the ‘high’ range for homocysteine [39,41]. Both these studies used at-risk participants.

All studies found an increase in B vitamin levels and reduction in homocysteine levels compared to the placebo (Table 4). This reiterates that B vitamins are readily absorbed in the blood, since this was evident even in the shorter trials of 28 days. The only exception to this was measures of folate, whereby three of the seven studies measuring folate failed to find a significant increase following supplementation.

Lastly, two studies considered whether any of these changes in micronutrient status could be associated with change in mood outcomes. One study found that changes in folate were not associated with either depression or anxiety [40]. Ford et al. [41] found poor correlations between vitamin B12, RC folate, and tHcy and a change in depressive symptoms. The heterogeneity in measures of nutrient status and mood precludes further analysis of changes in biomarkers in the context of clinical outcomes beyond the narrative description above and found in Table 4.

### 3.8. At-Risk Samples

Of the eight studies using ‘at-risk’ participants, five found a significant benefit for mood. Two of these studies conducted their own sub-group analysis in which they found differential effects with respect to baseline nutrient or mood status. Both Gosney et al. [40] and Hallert et al. [39] failed to find significant effects of supplementation in their full cohorts using the Hospital Anxiety and Depression Scale (HADS) and Psychological General Well-Being Index (PGWB), respectively. However, they found significant benefits of supplementation on both of these scales when analysing a subset of participants who scored abnormally or poorer at baseline.

## 4. Discussion

The present review of randomized controlled trials revealed that there is currently some evidence to suggest a positive effect of B vitamin supplementation for mood outcomes in healthy and at-risk adults. The present review was one of the few to examine both the broader measure of mood and its multi-faceted elements of depressive symptoms, anxiety symptoms, and stress independently. When examining the facets of mood, meta-analysis revealed that providing a supplement containing B vitamins provided a reduction in stress (*n* = 958, SMD = 0.23, 95% CI = 0.02, 0.45, *p* = 0.03). The reduction in depressive symptoms failed to reach statistical significance (*p* = 0.07) and there was no benefit to anxiety. Despite heterogeneity between the constituents across studies, most supplements exceeded recommended daily intakes of B vitamins (between 2 to 300 times). The examination of blood biomarkers revealed that no study included in this review reported a mean B vitamin status that was consistent with clinical deficiency at baseline. This may suggest a limited potential to observe mood effects despite supplementation levels well beyond the recommended daily intakes.

The analysis of effects on general mood and focusing on depression, anxiety, and stress proved a fruitful approach. While five of the nine studies that measured an overall measure of mood found benefits of B vitamin supplementation, analysis of subscales to examine mood facets captured specific domains of mood that may be sensitive to the B vitamin status, which may be missed in broad measures of mood. For example, the benefit of B vitamins was evident in more than half the studies that measured stress, while the benefits to depressive symptoms were less evident, with a significant effect over a placebo reported in only three of the eleven studies with this measure. This supports the finding from a previous review reporting the positive effects of multi-vitamin nutrients (some of which contained a high dose of B vitamins) for stress [8]. The previous review found the effect for subclinical depression failed to reach significance, which was similar to the findings of the pooled meta-analysis results of the present paper after excluding extreme outliers. To date, a gold standard measure of measuring mood in a healthy sample does not exist, as demonstrated by the heterogeneity of measures used in the included studies of this review. The findings of this review suggest that it may be worthwhile to utilise measures that provide an overall mood score as well as subscales, which may reveal particular mood facets that are more responsive to supplementation.

In general, a comparison across trials was difficult due to heterogeneity across supplement concentration, participants, and design. Only two of the 18 studies investigated B vitamins only, with the others using multi-vitamins. While there is evidence that B vitamins have some relationship with mood, the results of this review should be addressed with caution, given the possibility that the remaining constituents within each of the multi-vitamin/mineral formulas may have confounded any effects for mood. The strict criterion of at least 3 B group vitamins may have limited the studies selected, but was consistent with previous research supporting synergistic effects of combined B vitamins, to allow for nutrients to work in concert to modulate metabolic pathways [6]. Despite supplementing participants with B vitamins at levels that well exceeded their recommended daily intakes and at levels that could not reasonably be achieved through diet, the benefits were inconsistent across studies and mood facets.

A potential explanation for this pattern of improvement was revealed through the investigation of studies that utilized blood biomarkers to examine micronutrient status. While in almost all cases, the micronutrient levels significantly increased post-supplementation, the two studies that investigated the association between a change in micronutrient status and change in mood outcomes found poor correlations. Of the studies included in this review, none reported a mean B vitamin status that was considered clinically deficient. This was to be expected since most studies utilised healthy participants but is noteworthy when considering if the optimal nutrient status of participants in these trials, at baseline, may have limited the potential for detecting any mood effects [20]. Similarly, a study investigating the effects of folic acid in healthy males found no benefit for mood [26]. These authors suggest that if their participants had a deficiency in folic acid or diagnosis of a mood disorder at baseline, the benefits of supplementation may be more prominent [26]. More rigorous investigation is required to explain the discrepancy between observational studies in clinical groups and intervention studies in healthy populations.

Of the large number of studies investigating B vitamins, very few have been studied in healthy or at-risk populations, with research primarily confined to clinical groups. This may be a contributing factor as to why the majority of studies eligible for inclusion in the review contained many micronutrients and nutraceuticals. By including at-risk participants (who do not have a clinical diagnosis of a mood disorder), this review was able to provide a more comprehensive summary of this under-researched group. More than half of the studies included in this review that utilized at-risk samples found a benefit of supplementation for mood outcomes. The examination of subgroups within participants in clinical trials supports the suggestion proposed by Morris and Tangney [20] that the investigation of healthy populations will continually produce null results due to participant baseline nutrient levels already reaching an optimal level. Particularly, the benefit of studying at-risk groups was shown by two of the studies included in this review [39,40] who conducted their own sub-group analyses within their samples. Both studies found a beneficial effect of B vitamins in participants who had poor mood ratings at baseline, while there was no benefit when examining the entire sample. This finding is notable given that the methodology used within each study was consistent and, therefore, comparisons between participants are justified. This review supports further research of B vitamins for mood in at-risk groups. As highlighted by Walker, et al. [53], targeting individuals who have elevated levels of psychological distress or sub-clinical mood symptoms will capture individuals who may be overlooked in previous trials with ‘healthy’ individuals. There is also evidence to suggest a benefit of multi-nutrient supplementation for sub-clinical mood symptoms in healthy populations [8]. Novel recruitment strategies such as nation-wide mail-out invitations could be used to solicit sub-clinical individuals to participate in future studies. This is a group of participants who have previously been missed in previous research. 

Despite the link between diet and mood being a driving force for supplementation research, an important observation was that only two of the included studies considered food intake and dietary habits of their participants at baseline [43,46]. In addition, just under half of the studies measured changes in B vitamin biomarkers. Of these, only two studies attempted to examine associations between a change in vitamin status and a change in mood outcomes.

In order to further understand the processes behind biomarkers and shifts in micronutrient status, it is imperative that future studies measure baseline dietary habits as well as biomarker status [29]. It is highly important for increasing evidence to suggest that the relationship between individual nutrients and mood is confounded by overall diet quality [54]. Understanding a participant’s nutritional status across a range of markers at baseline will highlight if and in what aspects of diet, there is room for improvement [55]. Nutritional and supplementation research is slowly grasping that supplementing individuals who already have optimal nutrient status is not efficacious [29]. Rather, individuals with sub-optimal levels may be the most sensitive in intervention research [56].

An important caveat to consider in supplementation research is that supplementing with single nutrients can, by no means, mimic the complexity of diet [56]. There are many dietary factors and circulating levels of nutrients that are known to alter absorption of B vitamins, since medications can be a confounding factor in clinical research. Aspects of diet such as alcohol intake and omega-3s [57] should be considered in order to provide a more thorough understanding of dietary habits of trial volunteers and how nutrient status affects the response to intervention. Similarly, measuring methylmalonic acid and homocysteine may provide an insight into the mechanisms involved. 

The present review provides evidence for the benefit of B vitamin supplementation in healthy and at-risk populations for stress. The benefit for depressive symptoms failed to reach statistical significance (*p* = 0.07) and there was no benefit to anxiety. Given they are water soluble and well-tolerated, the potential for B vitamins to repair nutritional deficiencies not met through diet is promising [58] and offers a preventative approach to maintain mood. However, before a recommendation of B vitamins for mood benefits in healthy or at-risk populations can be given, further research with more rigorous methodologies is required [29]. In order to increase the sensitivity of future studies, researchers should consider utilizing participants who either (1) have poorer nutrient status at baseline, (2) have poorer mood outcomes at baseline, or (3) utilize a methodology that considers associations between changes in mood outcomes and changes in biomarker status to investigate this relationship.

Strengths of this review include conformity to the Preferred Reporting Items for Systematic Reviews and Meta-Analyses (PRISMA) method and breadth of literature searched, including consideration of participants who were ‘at-risk.’ Inclusion criteria guided by previous review and extension of Long and Benton [8] to look at facets of mood, not mood merely as one-dimensional was another benefit of this review. Despite this, the present review had the following limitations that should be considered. Trials were very heterogenous in methodology including sample populations, administered dosages of B vitamins, and supplementation periods. This may have contributed to the heterogeneity detected in the meta-analyses. Many of the studies utilized multivitamin/mineral supplements, which makes it difficult to isolate the effects to B vitamins in particular.

## 5. Conclusions

There is increasing consensus that nutrient status is an important modifiable factor in many neurological and psychiatric conditions. This present review provides evidence that B group vitamin supplementation (either alone or with a multivitamin) may also benefit mood in healthy and at-risk individuals. Further research utilising multiple B vitamins in at-risk groups (with suboptimal nutritional status or subclinical mood disturbances) is warranted. An increased understanding of how baseline micronutrient status and dietary habits influence the effects of supplementation will be essential in order to provide evidence-based recommendations on the benefit of B vitamins for mood.

## Figures and Tables

**Table 1:** Randomized, Double-Blind, Placebo-Controlled Trials investigating the effects of B vitamins on mood.

Study	Sample Size	Gender	Participant Characteristics	Age range (years)	Intervention	Length of intervention	Biomedical measures	Mood measures ^a^	Outcomes			
									Overall Mood	Depressive Symptoms	Anxiety Symptoms	Stress
Armborst et al. (2018)	*n* = 59 tx = 29 pb = 30	69% female	Chronic psychological stress	18–65	Multivitamin	12 weeks	N/A	PSQ, VAS				+
Camfield et al. (2013)	*n* = 138 tx = 56 pb = 60	56% female	Healthy	20–50	Multivitamin	16 weeks	B6, B12, RC folate, homocysteine	PSS				n.s
Carroll et al. (2000)	*n* = 80 tx = 40 pb = 40	100% male	Healthy	18–42	Multivitamin	28 days	N/A	GHQ, HADS, PSS, rating scales	+	n.s	+	+
Cockle et al. (2000)	*n* = 139 tx = 66 pb = 61	63% female	Healthy	60–83	Multivitamin	24 weeks	B1, B2, B6, folate, B12	POMS	n.s			
Ford et al. (2008)	*n* = 299 tx = 118 pb = 123*	100% male	Being treated for, or had a history of hypertension	> 75	B vitamins only	2 years	B12, RC folate, homocysteine	BDI		n.s		
Gosney et al. (2008)	*n* = 73 tx = 26 pb = 33	Not specified	nursing home residents	> 60	Multivitamin	8 weeks	Folate	MADRS, HADS		+ ^b^	n.s	
Hallert et al. (2009)	*n* = 65 tx = 28 pb = 29	61% women	coeliac patients	45–64	B-group vitamins only	6 months	Serum folate, B12, homocysteine	PGWB	+ ^c^	+ ^c^	+ ^c^	
Harris et al. (2011)	*n* = 56 tx = 25 pb = 25	100% male	sedentary occupation/little exercise	50–69	Multivitamin	8 weeks	N/A	GHQ, DASS, PSS, POMS, VAS	+ ^d^	n.s	n.s	n.s
Haskell et al. (2010)	*n* = 226 tx = 106 pb = 110	100% female	occasional subjective fatigue	25–50	Multivitamin	9 weeks	Hcy	Quality of life (SF36), CFS, POMS	n.s			
Kennedy et al. (2010)	*n* = 215 tx = 103 pb = 107	100% male	Healthy	30–55	Multivitamin	33 days	N/A	POMS, PSS, GHQ, Bond Lader, VAS	+ ^e^	n.s		+
Kennedy et al. (2011)	*n* = 215 tx = 94 pb = 104	100% male	Healthy	30–55	Multivitamin	28 days	N/A	Bond Lader, VAS				
Long and Benton (2013)	*n* = 101 tx = 43 + 41 pb = 42	100% male	Healthy	*M=* 20.9	Multivitamin	12 weeks	N/A	Picture frustration, Buss-Perry Aggression Scale, PSS				+
Macpherson et al. (2016)	*n* = 76 tx = 37 pb = 36	100% female	Healthy	50–75	Multivitamin	4 weeks	N/A	STAI, GHQ, HADS, Bond-Lader, VAS, PSS, CFS	n.s	n.s	n.s	n.s
Oliver-Baxter et al. (2018)	*n* = 50 tx = 22 pb = 28	100% female	Elevated psychological distress	25–45	Multivitamin	8 weeks	Folic acid	STPI		n.s	n.s	
Pipingas et al. (2013)	*n* = 138 tx = 56 pb = 60	56% female	Healthy	20–50	Multivitamin	16 weeks	N/A	GHQ, POMS, CFS, Bond-Lader, VAS, STAI	n.s	n.s	+ ^f^	
Schlebusch et al. (2000)	*n* = 300 tx = 151 pb = 149	Not specified	Highly stressed employees	18–65	Multivitamin	30 days	N/A	HARS, PGWB, VAS, BSI	+		+	+
Stough et al. (2011)	*n* = 80 tx = 20 + 22 pb = 18	66% female	Healthy	*M* = 42.2	Multivitamin	90 days	N/A	STAI, POMS, PSQ	n.s	n.s	n.s	+
White et al. (2015)	*n* = 58 tx = 28 pb = 30	50% female	Healthy	18–40	Multivitamin	4 weeks	B6, B12, folate, homocysteine	POMS, PSS, VAS, STAI	n.s	+	n.s	n.s

* Intention to Treat analysis included *n* = 138 per group. **+** indicates a statistically significant benefit to mood symptoms at the *p* < 0.05 level. n.s; not significant. ^a^ PSS: Perceived Stress Scale; GHQ: General Health Questionnaire; HADS: Hospital Anxiety and Depression Scale; POMS: Profile of Mood States; BDI: Beck Depression Inventory; MADRS: Montgomery-Åsberg Depression Rating Scale; PGWB: Psychological General Well-Being Index; DASS: Depression, Anxiety and Stress Scale; VAS: Visual Analogue Scales; CFS: Chalder Fatigue Scale; STAI: State Trait Anxiety Inventory; HARS: Hamilton Anxiety Rating Scale; PSQ: Personal Strain Questionnaire; STPI: State-Trait Personality Inventory. ^b^ Effect was only evident for individuals who had poor depression scores at baseline (*n* = 18). ^c^ Effect was only evident for individuals who had reduced psychological wellbeing at baseline (*n* = 23). ^d^ Effect was significant as measured by the Depression, Anxiety and Stress Scale and General Health Questionnaire, but no effect for Profile of Mood Scales. ^e^ Effect was significant as by General Health Questionnaire, but no effect for Profile of Mood Scales. ^f^ Effect was only evident in males (*n* = 51).

**Table 2:** Constituents of Supplements.

		B1 (mg)	B2 (mg)	B3 (mg)	B5 (mg)	B6 (mg)	B7 (µg)	B9 (µg)	B12 (µg)		
Other name		Thiamine	Riboflavin	Niacin, Nicotin- amide	Pantothenic acid		Biotin	Folate, Folic acid	Cyanocobalamin		
**Recommended Daily Allowance (19–50 years)**		1.1 (M) 1.2 (F)	1.3 (M) 0.9 (F)	16 (M) 14 (F)	5^	1.3	30^	400	2.4		
Study Daily Intake										No. of B vits	Other constituents of supplement
Camfield et al. (2013) Harris et al. (2011) Pipingas et al. (2013)	M	30	30	30	64.13	24.8	-	500	30	7	Vitamins C, D, E, Choline, Calcium, Iron, Magnesium, Selenium, Zinc, Korean ginseng, Siberian ginseng, Ginkgo Biloba, Chamomile, Green Tea, St. Mary’s thistle
	F	50	50	50	68.7	41.14	-	500	50		
Carroll et al. (2000) Kennedy et al. (2010) Kennedy et al. (2011)		15	15	50	23	10	15	400	10	8	Vitamin C, Calcium, Magnesium, Zinc
Armborst et al. (2018)		25	25	100	100	25	-	800	50	7	Taurine, L- ornithine, L-phenylalanine, L-tyrosine, Vitamin C, ß-carotene, Magnesium, Zinc, Selenium, Chrome, Molybdenum
Cockle et al. (2000)		14	16	180	-	22	-	4000	30	6	Vitamins A, C
Ford et al. (2008)		-	-	-	-	25	-	2000	400	3	N/A
Gosney et al. (2008)		4.8	5.6	56	20	12	120	2400	800	8	Vitamins A, C, D, E
Hallert et al. (2009)		-	-	-	-	3	-	800	500	3	N/A
Haskell et al. (2010)		4.2	4.7	54	18	6	-	600	3	7	Vitamins A, C, D, E, K1, Calcium, Phosphorus, Chromium, Copper, Fluoride, Iodine, Iron, Magnesium, Manganese, Molybdenum, Selenium, Zinc
Long and Benton (2013)		1.4	1.75	20	7.5	2	62	200	2.5	8	Vitamins A, C, D, E, K, calcium, phosphorus, magnesium, potassium, chloride, iron, iodine, copper, manganese, chromium, molybdenum, selenium, zinc, lutein.
Macpherson et al. (2016)		30	30	20	70	30	150	500	115	8	Vitamins A, C, D, E, K, Zinc, Calcium, Magnesium, Selenium, Molybdenum, Chromium, Manganese, Iron, Copper, Iodine, L. rhamnosus, L. acidophilus, Bifidobacterium longum, Citrus bioflavonoids extract, cranberry Pacran, St. Mary’s thistle, damiana, skullcap, grape seed, nettle, Coenzyme Q10, globe artichoke, black cohosh, turmeric, ashwagandha, hawthorn, silicon, Bacopa monnieri, Lecithin, Spearmint oil, bilberry, marigold
Oliver-Baxter et al. (2018)		12.5	12.5	25	37.5	25	37.5	150	25	8	Vitamin C, Magnesium oxide, Zinc, Withania somnifera
Schlebusch et al. (2000)		15	15	50	23	10	150	-	10	7	Vitamin C, Calcium, Magnesium
Stough et al. (2011)		75	10	100	68.7	25	20	150	30	7	Vitamins C, E, Calcium, Magnesium, Potassium, Avena sativa, Passion flower, Lecithin, Choline, Inositol
White et al. (2015)		18.54	15	50	23	10	15	400	10	7	Vitamin C, Calcium, Magnesium, Zinc

Composition of supplements. M; male, F; female. Recommended Daily Allowance (RDA) values obtained from Institute of Medicine 1998 [48]. RDAs are set to meet the needs of almost all (97 to 98 percent) individuals in the 19–50 years group. ^indicates Adequate Intakes (AIs) are reported instead of RDA. AIs is used as an alternative as there is uncertainty in the data to specify the percentage of individuals covered by this intake.

**Table 3:** Baseline biomarker status.

Study	Group	Vitamin B6	Folate (RC)	Folate (Serum)	Vitamin B12	Homocysteine
Armborst et al. (2018)	Placebo	-	-	9.48 (3.08)	-	-
	Active	-	-	9.67 (4.13)	-	-
Camfield et al. (2013)	Placebo	114.08 (10.27)	944.96 (21.94)	-	314.60 (14.48)	10.6137 (0.22)
	Active	111.70 (9.36)	933.27 (34.99)	-	289.25 (12.11)	10.8444 (0.35)
Cockle et al. (2000)	Placebo Males	235.64 (75.74)	-	9.74 (5.66)	304.10 (160.93)	-
	Placebo Females	239.02 (54.95)	-	9.31 (4.13)	323.44 (150.06)	-
	Active Males	243.74 (50.06)	-	8.63 (3.70)	307.26 (86.29)	-
	Active Females	230.72 (46.85)	-	9.10 (4.76)	365.66 (156.38)	-
Ford et al. (2008)	Placebo	-	-	24.22 (7.44)	253.30 (115.10)	13.06 (3.83)
	Active	-	-	24.00 (7.50)	253.10 (107.50)	13.59 (4.43)
Gosney et al. (2008)	Overall	-	-	5.88 (range 0.9–39.8)	-	-
Hallert et al. (2009)	Placebo	-	-	14.8 (range 7.7–23.4)	431 (174–734)	11.4 (7.4–21.9)
	Active	-	-	12.9 (range 6.7–44)	326 (141–510)	11.70 (7.8–23.0)
Haskell et al. (2010)	Placebo	-	-	-	-	10.93 (0.53)
	Active	-	-	-	-	10.94 (0.46)
Oliver-Baxter et al. (2018)	Placebo	-	-	27.1 (13.9)	-	-
	Active	-	-	28.2 (16.7)	-	-
White et al. (2015)	Placebo	85.00 (34.02)	944.25 (190.09)	-	304.88 (75.34)	11.43(2.24)
	Active	84.95 (23.73)	954.63 (188.58)	-	301.04 (82.22)	11.78 (1.81)

Standard deviation/error reported in parentheses.

**Table 4:** Change in biomarkers in response to supplementation.

	Vitamin B1	Vitamin B2	Vitamin B6	Vitamin B12	Folate (RC)	Folate (serum)	Homocysteine
Armborst et al. (2018)						+	
Camfield et al. (2013)			+	+	+		-
Cockle et al. (2000)	+	+	+	+		+^	
Ford et al. (2008)				+		+	-
Gosney et al. (2008)						+^	
Hallert et al. (2009)				+		+	-
Haskell et al. (2010)							-
Oliver-Baxter et al. (2018)						+	
White et al. (2015)			+	+	+		-

**+** indicates a statistically significant increase at the *p* < 0.05 level, **+^** indicates an increase that was not statistically significant, **-** indicates a statistically significant decrease at the *p* < 0.05 level.

**Figure A1:**
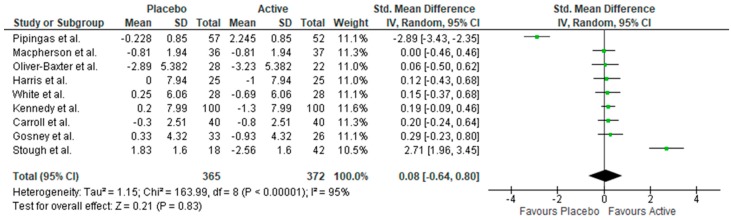
Depressive symptoms with outliers included.
